# Adolescent girls’ perceptions regarding the use of contraceptives in Ekurhuleni District, Gauteng

**DOI:** 10.4102/hsag.v29i0.2580

**Published:** 2024-07-24

**Authors:** Pretty T. Moloi, Rakgadi G. Malapela

**Affiliations:** 1 Department of Health Studies, Faculty of Human Sciences, University of South Africa, Pretoria, South Africa

**Keywords:** adolescent girls, contraceptive use, Ekurhuleni, perceptions, unintended pregnancy

## Abstract

**Background:**

Unintended pregnancies pose various challenges for adolescent girls and can be prevented through contraceptive use. However, contraceptive use among adolescents is lower compared to among older women.

**Aim:**

To describe adolescent girls’ perceptions of the use of contraceptives.

**Setting:**

Two high schools in Tembisa were selected as the study setting.

**Methods:**

Purposive sampling was used to select adolescent girls who had reached menarche and were willing to participate. Data collection involved narrative writing for girls under 16 and interviews for older adolescents. Braun and Clark’s six-step method was used for data analysis.

**Results:**

A total of 35 adolescent girls were identified, and the final sample size was *N* = 33 adolescent girls whose ages ranged from 13 to 18 years. Two main themes emerged. Theme 1 was positive perceptions of contraceptives. Theme 1 was supported by two subthemes: the first was the influence of the male partner; the second was financial constraints in families. The second theme was negative perceptions of contraceptives. Four subthemes supported the second main theme, which were: myths and misconceptions, influence of parents, healthcare worker attitudes and negative attitudes of community members.

**Conclusion:**

Addressing the low contraceptive uptake among adolescent girls requires understanding their perceptions to tailor interventions accordingly.

**Contribution:**

The study contributes by highlighting the negative perceptions of contraceptive use, including family financial constraints and the influence of male partners. These findings can inform reproductive health initiatives aimed at increasing contraceptive use among adolescents.

## Introduction

Contraceptives are procedures, medications, behaviours or devices that prevent pregnancy among women (Bansode, Sarao & Cooper 2023). The use of contraceptives enables women to prevent unintended pregnancies, giving them control of reproductive choices. It is estimated that at least two of three women aged 15–49 years use contraceptives globally; however, rates of usage vary with the lowest rate of 15% being among adolescent girls aged 15–19 years in sub-Saharan Africa including South Africa (Chola, Hlongwana & Ginindza 2023). The low usage of contraceptives among adolescent girls in sub-Saharan Africa is because of several reasons such as a lack of knowledge and access (World Health Organization [WHO] 2023). In South Africa, 31% of adolescent girls had an unmet contraceptive need by the end of 2016 (Govathson et al. 2023). As a result of the low usage of contraceptives among adolescent girls, there is a high level of unintended pregnancies, with at least 25% of adolescent girls having unintended pregnancies in South Africa (Govathson et al. 2023). Unintended pregnancies are associated with several challenges among adolescent girls. These include unsafe abortion, mental illness, school dropouts, pregnancy and childbirth-related complications and death (Olika et al. 2021). Among adolescents, complications related to pregnancy and childbirth are the leading cause of death (Olika et al. 2021).

Persons from the ages of 10 to 19 years are classified as adolescents (United Nations International Children’s Emergency Fund [UNICEF] 2018). Adolescence is a transition phase from childhood to adulthood characterised by physical and emotional growth, which also includes sexual maturation (Bukenya et al. 2020). As adolescents mature sexually, there is a tendency to engage in risky sexual practices such as unprotected sex resulting in unintended pregnancies, which could be prevented by contraceptive use (Bukenya et al. 2020). In South Africa, from the age of 12 years, adolescent girls can access reproductive health services including contraceptives without parental and/or guardian consent (*Children’s Act of 2005*). In public health facilities, contraceptives are also provided free of charge to enable access for everyone including adolescents (Kriel et al. 2023).

Several types of contraceptives are available for use by adolescents, and these include long-acting reversible contraceptives (LARC), emergency contraceptives, female and male sterilisation, natural methods and barrier methods (Chimurkar et al. 2021). Long-acting reversible contraceptives provide long-term pregnancy prevention and are highly effective and include implants, intrauterine devices (IUDs) and injectables (Chimurkar et al. 2021). The etonogestrel implant is regarded as the most effective contraceptive (Bansode et al. 2021). Despite the effectiveness of LARC, among adolescents in sub-Saharan Africa using contraceptives, only 29.8% of them use LARC, with most preferring injectables (McCurdy, Jiang & Schnatz 2018). Some of the LARCs form part of hormonal contraceptives such as the Levonorgestrel intrauterine system, injectables and patches. In South Africa, a survey in Cape Town found that 63.7% of adolescents use hormonal contraceptives (Toska et al. 2020).

Another common contraceptive choice used by adolescent girls are the barrier methods, which include male and female condoms, cervical caps and diaphragms used with spermicides (Hassoun 2018). One important advantage of the barrier methods is that they offer protection from sexually transmitted infections (STIs) including HIV. A 2017 South African survey found that the highest condom use was among young people aged 15–24 years (Duby et al. 2021). This rate of condom use among young people in South Africa is below optimum levels in comparison to the USA where at least 81% of young people use condoms (Barrett et al. 2021; Duby et al. 2021). In addition, since 2017, the number of young people using condoms in South Africa has been declining annually (Duby et al. 2021). Among adolescent girls aged 15–19 years, at least 55% use condoms in South Africa (Davids et al. 2021). This sub-optimal level of condom utilisation among adolescent girls increases their risk of unwanted pregnancies and contracting STIs including HIV (Beksinska, Wong & Smit 2020). Although the use of condoms is higher among adolescent girls relative to other contraceptive methods, the failure rate of condoms is also high because of incorrect and inconsistent use (Ayalew et al. 2022; Sharma & Singh 2022).

Considering the availability of several contraceptive choices and their right to access contraceptives, adolescent girls are less likely to use a contraceptive at sexual debut and only use it after they have fallen pregnant (Sharma & Singh 2022). Among 17 African countries assessed, South Africa had the highest (70%) rate of contraceptive use among adolescents who had previously fallen pregnant (Ahinkorah et al. 2021). There is consensus that adolescent girls are aware of contraceptives although knowledge of specific methods of contraceptives is inadequate, which affects how they use contraceptives (Sharma & Singh 2022; Gyan & Marhefka-Day 2021; Jonas et al. 2020). In the South African study, adolescent girls reported that they needed more information on contraceptives and the majority knew of injectables, a few mentioned implants and no other contraceptive was mentioned (Jonas et al. 2020). Associated with the lack of knowledge are misconceptions and myths about contraceptives. In a systematic review of studies conducted among adolescents from low to medium-income countries, it was concluded that myths about contraceptives contributed to the low usage of contraceptives (Munakampe, Zulu & Michelo 2018). A South African country-wide study also concluded that misconceptions about contraceptives determine use, and these misconceptions were based on choices of contraceptives (Pleaner et al. 2022). For example, adolescents perceived that pills and injectables are associated with future infertility and weight gain, implants were misconceived to rust in the body, and IUDs were placed in the vagina and fill it up (Pleaner et al. 2022).

Interpersonal relationships including intimate partners and parents also influence adolescent girls’ use of contraceptives (Kriel et al. 2019). A study in Ghana found that the nature of sexual relationships and cultural norms affect the use of contraceptives among adolescent girls (Gyan & Marhefka-Day 2021). A South African study explained the issue of power in influencing the use of contraceptives in sexual relationships between men and adolescent girls (Duby et al. 2023). Findings from the study highlight that although there is a shift from traditional gender role expectations, there is still power inequity in sexual relations with men determining the use of contraceptives, especially condoms (Duby et al. 2023). Although men exert power over the use of contraceptives, their power does not deny adolescent girls from negotiating the use of contraceptives including condoms (Duby et al. 2023). Notably, male partner influence is regarded as the most significant interpersonal factor affecting the use of contraceptives among adolescent girls (Kriel et al. 2019). Parents also influence adolescent girls’ use of contraceptives (Chola et al. 2023). Parents can either encourage or discourage adolescent contraceptive use (Chola et al. 2023). On one hand, parents often discourage adolescent girls from using contraceptives assuming their children are engaging in sexual intercourse when they are still too young (Jonas et al. 2020). On the other hand, parents encourage the use of contraceptives for adolescent girls who would have previously had an unintended pregnancy (Jonas et al. 2020).

Healthcare workers are also influential in the use of contraceptives among adolescent girls. Negative attitudes from healthcare workers discourage the use of contraceptives among adolescent girls (Jonas et al. 2020). Such negative attitudes are described in various contexts such as Zambia and South Africa and include rudeness, unfriendly facial expressions and asking unsettling questions like ‘Are you not too young for contraceptives?’ (Chola et al. 2023; Jonas et al. 2020). In addition, adolescents perceive that there is a lack of privacy when seeking contraceptives; for example, nurses will loudly ask if they are seeking contraceptives, which is a hindrance to contraceptive use (Jonas et al. 2020).

In low- and middle-income countries, which include South Africa, contraceptive use among adolescent girls is also affected by financial issues pertaining to the cost of travel for services and the cost of contraceptives (Li et al. 2020). In addition to the inability to afford transport costs associated with accessing contraceptives, adolescent girls in low- to middle-income countries do not always have the freedom to travel to healthcare centres as they please (Chandra-Mouli & Akwara 2020). In South Africa, the issue of distance to clinics is also highlighted particularly in rural areas where adolescents must travel long distances, and parents do not always have money to pay for the travel (Jonas et al. 2020).

The study used the social ecological model (SEM). The SEM assumes there is an interaction of four levels that are related that affect the use of contraceptives. These levels include the intrapersonal, interpersonal, community and societal levels (Bronfenbrenner 2005). At the centre are the intrapersonal factors such as knowledge, myths and misconceptions about contraceptive use. The next level is the interpersonal level that includes relationships with partners, peers and parents. The third level is the community level that includes interactions with community members and services in the community like clinics and religious organisations. The last level is the society level that comprises the policies in the broader society affecting the use of contraceptives such as allowing adolescents to access contraceptives without parental consent.

The study was conducted in the Ekurhuleni district in Gauteng Province. Ekurhuleni district is one of the three metropolitan municipalities in Gauteng Province. In South Africa, Gauteng province has the highest number of teenage pregnancies, and within the Ekurhuleni district, at least 30% of adolescent girls have had an unintended pregnancy (Nkosi & Pretorius 2019). The higher prevalence of adolescent pregnancies in the Ekurhuleni district compared to the national prevalence of 25% is concerning; therefore, there is a need to understand the use of contraceptives among adolescent girls. Previous studies on contraceptive use among adolescent girls have been conducted in contexts outside Ekurhuleni; for example, Jonas et al. (2020) conducted their study in Tshwane and provinces outside Gauteng; Hlongwa, Tlou and Hlongwana (2020) also conducted their study in Umlazi Township in KwaZulu-Natal. The study conducted in Ekurhuleni by Pleaner et al. (2020) focussed on adolescents enrolled in a reproductive health intervention. This study complements these previous studies by providing adolescent girls’ perceptions in Ekurhuleni on contraceptive use where unintended teenage pregnancies are higher than the national prevalence. Understanding the perceptions of adolescents in this context would facilitate the development of reproductive health programmes to address the problem of inadequate contraceptive usage at intrapersonal, interpersonal, community and societal levels. Therefore, the purpose of the study was to describe perceptions of adolescent girls regarding the use of contraceptives in Ekurhuleni District, Gauteng.

## Research methods and design

The qualitative approach with a descriptive study design was used. Likewise, this study seeks to expand existing knowledge on adolescent girls’ use of contraceptives from previous studies conducted in South Africa.

The study was conducted in Tembisa, which is the second-largest township in South Africa and is in the City of Ekurhuleni (Department of Cooperative Government and Traditional Affairs [COGTA] 2021). The study was conducted at two high schools in Tembisa. Within the Ekurhuleni district, the population of adolescents is approximately 490 702 (13% of the total population) (COGTA, 2021). The inclusion criteria were all adolescent girls aged between 13 and 19 years who had reached menarche and gave consent or whose parents consented on their behalf to participate in the study for participants younger than 16 years. For these participants younger than 16 years, in addition to their parents consenting on their behalf, they individually consented by signing an assent consent form. The researcher determined the inclusion of adolescent girls who had reached menarche, by first asking the prospective participants. A total of 35 prospective participants were identified. The exclusion criteria were adolescent girls aged 13–19 years who had not reached menarche and/or who did not consent to participate in the study. The researcher P.T.M. recruited the study sample in April 2023 in Tembisa.

Convenience sampling was used to select the two high schools. Convenience sampling refers to a non-probability sampling technique that entails the selection of elements readily available to the researcher (Polit & Beck 2020). Purposive sampling was done to select the sample of adolescent girls who participated in the study. Purposive sampling makes use of the researchers’ judgement to select participants (Polit & Beck 2020). In this case, the judgement was based on the age of the adolescent girls. The sample size was determined by data saturation, which occurs when additional sampling does not provide new information (Grove & Gray 2019).

Data were collected using written narratives and interviews by the researcher P.T.M. Narratives entail the writing of words to explain a situation in the way the writer visualises it (Habibi et al. 2020). Participants were given small notebooks that were labelled with codes and wrote responses to questions on contraceptive use. The questions included: What are your thoughts regarding contraceptive use? What are your experiences regarding contraceptive use? What factors do you think promote contraceptive use? What challenges do you experience when you want to use contraceptives? What factors prevent you from using contraceptives? What do you think can be done to promote contraceptive use? The notebooks were collected after 1 week. Data collection through written narratives was done for younger participants (˂ 13 years) who were shy to express their perceptions through interviews. Participants wrote their narratives in English or IsiZulu. Notably, the researcher P.T.M. who collected data is fluent in both English and IsiZulu.

Interviews were conducted for older adolescents aged more than 16 years. Interview questions were exactly like questions in the written narratives. The interviews were conducted at the two high schools in Tembisa in a classroom not in use, which afforded privacy during the interviews. A semi-structured interview guide was used during the interviews. The interview guide was pretested with three adolescent girls, and the results were not used in the final analysis. The purpose of pretesting the interview guide was to check its effectiveness. Interviews lasted 30 min – 60 min and were audio recorded. Interviews were conducted in English or isiZulu. Data collection was conducted for 3 months from April to June 2023.

Data analysis was done using Braun and Clark’s six steps of thematic analysis (Clarke & Braun 2017). The first step was data familiarisation, whereby the researchers read the narratives and transcribed and translated the data from the interviews. Back translation was also done to ensure the accuracy of translated data. The second step was generating the initial codes. Open coding was used, which meant there were no preconceived codes, but codes were generated as the analysis was conducted. Interesting pieces from the data aligned to the research objective were grouped and assigned a code; an example of interesting data assigned a code was ‘fear of parents’. The third step was to generate themes by assembling all the collected information, placing it into categories, themes and subthemes. The fourth step was to review the themes, by reading the initial themes generated and aligning all remaining data to the new themes. This ensured that all data supported the themes generated. The fifth step was defining the generated themes, and the sixth was presenting the findings.

Throughout the data collection and data analysis, the researchers reflected on the distinction of their roles as researchers and previous experience to ensure data collected and analysed remains objective. This reflection considered the role of P.T.M. as a healthcare worker in a public healthcare facility and R.G.M. as an academic.

Trustworthiness was ensured by using the framework developed by Kyngäs, Kääriäinen and Elo (2020); (Lincoln & Guba 1985). Credibility was ensured by prolonged engagement with the participants, and integration of the data was made into the research findings. Dependability was ensured using a co-coder. Confirmability was ensured by checking and rechecking the developed themes, which would enable other researchers to draw similar conclusions should the research be repeated. In addition, to ensure confirmability, a description of the research design and methods was provided. To ensure transferability, the research setting and participants have been described in detail.

### Ethical considerations

Ethical permission to conduct the study was obtained from the University of South Africa Human Sciences Ethical Review Committee. The reference number is 15750523_CREC_CHS_2022. Permission to conduct the study was also granted by the Gauteng Province Department of Education and the two high schools where the study was conducted. Pseudonyms were used to protect the identity of participants and participants were assured that all information they provided would not be traced to them and would be used solely for the study. To ensure participants were protected from harm, such as physical or emotional discomfort, they were informed that they could tell the researcher to stop the interview at any time.

## Results

### Sample demographic characteristics

A total of 33 participants constituted the sample. All participants were female with 21 (63.6%) of participants from high school A and another 12 (36.4%) from high school B. The interviewed participants comprised 48.5% (*n* = 16) while those who provided narratives comprised 51.5% (*n* = 17). Most participants, that is, 24 (72.7%) were not in a relationship, while 9 (27.3%) were in a relationship. One participant had a child and one was pregnant at the time of data collection. Participants’ ages ranged from 13 to 18 years, and most, 24.2% (8) participants were 17 years. Most participants (88%) did not use contraceptives, while 6% were currently using contraceptives, and 6% had previously used contraceptives. Table 1 shows the sample demographic characteristics.

**TABLE 1:** Sample demographic characteristics.

Participant	High school	Interview or narrative	Age (years)	Relationship status	Has a child or pregnant	Use of contraceptives
1	A	Interview	16	Yes	No	No
2	A	Interview	17	No	No	Yes
3	A	Interview	16	No	No	No
4	A	Interview	16	Yes	Yes	Yes
5	A	Interview	16	Yes	No	Previously used
6	A	Interview	16	Yes	No	No
7	A	Interview	17	No	No	No
8	A	Interview	18	Yes	No	No
9	A	Interview	17	No	No	No
10	B	Interview	17	No	No	No
11	B	Interview	18	Yes	No	No
12	B	Interview	17	Yes	No	Previously used
13	B	Interview	17	Yes	No	No
14	B	Interview	17	No	No	No
15	B	Interview	18	Yes	Pregnant	No
16	B	Interview	17	No	No	No
17	A	Narrative	13	No	No	No
18	A	Narrative	13	No	No	No
19	A	Narrative	15	No	No	No
20	A	Narrative	13	No	No	No
21	A	Narrative	14	No	No	No
22	A	Narrative	14	No	No	No
23	A	Narrative	14	No	No	No
24	A	Narrative	14	No	No	No
25	A	Narrative	15	No	No	No
26	A	Narrative	13	No	No	No
27	A	Narrative	15	No	No	No
28	A	Narrative	15	No	No	No
29	B	Narrative	15	No	No	No
30	B	Narrative	13	No	No	No
31	B	Narrative	13	No	No	No
32	B	Narrative	15	No	No	No
33	B	Narrative	15	No	No	No

### Findings on perceptions of contraceptive use among adolescent girls

The participants described positive and negative perceptions of contraceptive use. The positive perceptions included the prevention of pregnancy when male partners do not support the use of condoms, and pregnancy prevention when there are financial constraints in families. Negative perceptions of contraceptive use were associated with myths and misconceptions, fear of parental retribution, healthcare worker judgemental attitudes and community member attitudes. The themes and subthemes are summarised in Table 2.

**TABLE 2:** Summary of themes and subthemes.

Themes	Subthemes
1. Positive perceptions of contraceptives	1.1. Influence of the male partner 1.2. Financial constraints in families
2. Negative perceptions of contraceptive use	2.1. Myths and misconceptions of contraceptive use 2.2. Parental influence 2.3. Healthcare worker attitudes 2.4. Community members’ judgemental attitudes

### Theme 1: Positive perceptions of contraceptives

The participants shared their positive perceptions of contraceptive use. Two subthemes supported the positive perceptions of contraceptive use. The first subtheme was the influence of the male partner, and the second was the economic constraints in families.

#### Subtheme 1.1: Influence of the male partner

The influence of the male partner in contraceptive use was discussed by participants who noted how contraceptives are useful when their male partners are not forthcoming to use condoms. In addition, in some relationships that are abusive, contraceptives were perceived to be positive for pregnancy prevention when condoms cannot be used. The quotes to support the subtheme are shown below:

‘Mostly boys don’t want to be having kids, so they send their girlfriends or their sexual partners to have these contraceptives because the boys don’t want to use condoms or protection.’ (Participant 15, from high school B, 18 years)

Participant 6 from high school A explained the influence of male partners especially when adolescent girls are in abusive relationships and the male partner does not want a baby. The quote is shown below:

‘Sometimes they avoid being pregnant … and maybe they are in an abusive relationship and the partner that they are in a relationship with doesn’t want babies. So, they try by all means to avoid terminating the pregnancy.’ (Participant 6, From high school A, 16 years)

While the use of contraceptives is generally positive, the above findings add a reality that they could be used as tools of compromise in abusive relationships. This also highlights reproductive rights weaknesses among adolescent girls and the desperate rather than elective use of contraceptives. This suggests an important empowerment dimension that adolescent girls lacked in sexual relationships.

#### Subtheme 1.2: Financial constraints in families

The participants also described how it was good to use contraceptives to prevent pregnancy because they came from families that were currently facing financial constraints and could not afford to support another child. Therefore, because of such financial hardships, the use of contraceptives was perceived as positive to prevent an unintended pregnancy. The quotes from participants are shown below:

‘I think it’s good because without the contraceptive pills or anything a lot of teenage girls would be pregnant by now … and would not know what to do with the baby due to their circumstances at the home of not being able to support the baby.’ (Participant 16, from high school B, 17 years)

‘It’s a good thing because like many families are in situations that are not right so bringing another baby to the family will be a burden to the parents.’ (Participant 2, from high school A, 17 years)

From the participants’ shared perceptions of contraceptive use, it was concluded that the positive perceptions of contraceptive use were influenced at an interpersonal level by relationships with partners. The financial constraints experienced in families also influenced a positive perception of contraceptives at the interpersonal level of the SEM.

### Theme 2: Negative perceptions of contraceptive use

The participants described the negative perceptions they had of contraceptive use. Four subthemes supported the theme. The subthemes were myths and misconceptions regarding contraceptive use, healthcare worker attitudes, attitudes of community members and negative parental influence.

#### Subtheme 2.1: Myths and misconceptions of contraceptive use

Participants described various myths and misconceptions that influenced their negative perceptions regarding contraceptive use. These misconceptions included that contraceptives cause weight gain and future infertility, and one participant noted that implants that are not placed correctly can have a systematic effect on the body. Quotes to support the subtheme are shown below:

‘It’s bad because it changes your weight and sometimes you look like a different person because it changes your appetite, you no longer eat the way you used to. You just become a different person.’ (Participant 13, from high school B, 17 years)

In addition to weight changes, participants also perceived that contraceptives were bad because of a misconception of causing future infertility:

‘The bad thing about using contraceptives is that in the long run, you might not be able to conceive, or it changes your body like the example, weight loss, gain loss, weight loss and gaining weight.’ (Participant 12, from high school B, 17 years)

The participants also described how early use of contraceptives could result in an inability to carry a pregnancy to term:

‘I had a family member who lost their child last year, when she was 7 months pregnant and because she has been using contraceptives for the longest time and when she finally had the chance to have a child that was taken away from her.’ (Participant 10, from high school B, 17 years)

As a result of the misconceptions that brought about negative perceptions of contraceptives, participants described how they would use contraceptives only after having a baby. The excerpt from Participant 15 is shown below:

‘My perception is it is a bad thing, the reason being is that most girls use it and end up that they can’t have kids or carry a baby to full term. Based on that, that’s what I think and have told myself that I would never take it, I would have my first kid and then I would go for contraceptives.’ (Participant 15, from High School B, 18 years)

#### Subtheme 2.2: Parental influence

Participants also noted the negative consequences of contraceptives such as that if parents found out it would imply to parents that their adolescent children were engaging in sexual relationships, which was not approved by their parents. The quotes from participants are shown below:

‘Parents are, sometimes so strict and sometimes the situation in the house is not good.’ (Participant 9, from high school A, 17 years)

‘The fear of parents finding out, that they might be intimate, and some might go back home so they are avoiding things such as those.’ (Participant 12, from high school B, 17 years)

In addition to fear of parents, another participant also illustrated how parents fuel myths and misconceptions on contraceptive use, which resulted in adolescents having a negative perception of contraceptives. The quote below supports the subtheme:

‘My parents don’t know that I am in a relationship and that I am having sexual intercourse and so to prevent it, it’s a lot of job. My mother, I don’t think she would like that, like as young as I am I’m having sexual intercourse and I have to prevent it. According to them, they think that prevention is not good it damages the body because at some point going forward, it may damage your womb from giving birth.’ (Participant 1, from high school A, 16 years)

#### Subtheme 2.3: Healthcare worker attitudes

The interaction with healthcare workers with judgemental attitudes was another issue that elicited negative perceptions of contraceptive use described by the participants. Participants highlighted that health workers perceived that they were too young to be using contraceptives. Participants’ quotes supporting the subtheme are shown below:

‘Many young kids are falling pregnant because when we go to clinics, we are told we are too young to make such decisions.’ (Participant 14, from high school B, 17 years)

‘When they arrive at the clinic, they are being judged by the nurses or like other people. Yeah, they’ll be telling them that like they are too young to use these contraceptives. So, that can prevent a person from using contraceptives.’ (Participant 8, from high school A, 18 years)

#### Subtheme 2.4: Community members’ judgemental attitudes

The participants shared negative perceptions of contraceptive use because being seen in queues for contraceptives at local clinics elicited judgemental attitudes from community members, including neighbours. Participants shared how they were concerned about what community members would think of them. The quotes to support the subtheme are shown below:

‘Comments of people and nurses everybody knows that when you by the family planning line you sleeping with someone.’ (Participant 30, from high school B, 13 years)

‘And another factor is that I would not want people to see me in ques err, what’s this. Family planning queues, because they will say that I am too young to be standing there.’ (Participant 13, from high school B, 17 years)

‘I think some people can be judgemental when you are going to the clinic. Maybe neighbour you find at the clinic and they will be judgemental.’ (Participant 5, from high school A, 16 years)

The adolescent girls’ negative perceptions of contraceptive use because of myths and misconceptions reflected the intrapersonal level of the SEM. Parents, in turn, illustrated the influence of interpersonal factors on the use of contraceptives among the adolescents in Ekurhuleni. The interactions with healthcare workers and community members demonstrate the community-level factors affecting contraceptive use.

## Discussion

The study found that adolescent girls had positive and negative perceptions regarding the use of contraceptives. The positive perceptions were that contraceptives were good in cases where condoms could not be used. They were also good for preventing unintended pregnancies in families that cannot afford to support another child. The negative perceptions were founded on myths and misconceptions such as weight gain and future infertility, which impelled them to use contraceptives after having a child. Negative perceptions of the use of contraceptives were also founded on the fear of being caught in possession of such items by parents. The study also found that adolescent girls perceived contraceptive use negatively because of healthcare workers’ and community members’ judgemental attitudes.

The influence of male sexual partners is described in studies across various contexts. The study by Kriel et al. (2019) found that the male partner influence is the strongest influence on contraceptive use among adolescent girls. In South Africa, Duby et al. (2023) also describe the power men exert over contraceptive use among adolescents. In this current study, the influence of the male partner confirms the findings by Duby et al. (2023) and Kriel et al. (2019); male dominance is described by Participant 15 who notes: ‘Boys don’t want to have kids, so they send their girlfriends or their sexual partners to have these contraceptives’. Despite the dominance, illustrated by girls being told to use contraceptives by male partners, participants in this study highlighted that they perceived this positively as it prevented pregnancy among adolescent girls through enabling contraceptive use. Given these findings, it is critical to acknowledge the role of the male partner in increasing contraceptive use among adolescent girls.

Adolescent girls in the study also demonstrated that they were not oblivious to the financial constraints that their families endure. In this regard, they held positive perceptions of contraceptives as a means of preventing unintended pregnancies to prevent additional financial burden to their families. Studies previously conducted discuss the issue of financial constraints in families as a hindrance for adolescent girls to access contraceptives as they cannot afford costs associated with accessing contraceptives such as transport costs (Li et al. 2020). Notably, the study conducted by Li et al. (2021) draws these conclusions from 103 low- to middle-income countries using data from demographic health surveys. In South Africa, the study conducted by Kriel et al. (2023) acknowledges that contraceptives are provided free of charge in public health facilities; however, financial challenges are related to transport costs associated with accessing the contraceptives. The difference in conclusion between our study and the studies conducted by Kriel et al. (2023) and Li et al. (2021) could warrant further studies to ascertain how financial constraints in families influence the use of contraceptives.

Several negative perceptions of the use of contraceptives were also discussed by the adolescent girls. The study confirmed previous findings by Pleaner et al. (2022) regarding the presence of myths and misconceptions surrounding contraceptive use, which contributed to a negative perception of contraceptives. Participants described how contraceptive use resulted in future infertility:

‘I would have my first kid and then I would go for contraceptives.’ (Participant 15, from high school B, 18 years)

This assertion by Participant 15 shows the strong influence of misconceptions on contraceptive use. In addition, the use of contraceptives only after having a child also reflects on findings of the study conducted in India by Sharma and Singh (2022) who found that adolescent girls only made use of contraceptives after having a child. Furthermore, Ahinkorah et al. (2021) found that South Africa has a high number of adolescent girls who use contraceptives only after having a first child among 17 countries assessed. The misconceptions and consequent negative perceptions on contraceptive use also illustrate the lack of knowledge on contraceptive use described by Sharma and Singh (2022).

The attitudes of healthcare workers and community members also influenced negative perceptions of contraceptive use. The issue of negative judgemental attitudes from community members and healthcare workers confirms findings by Jonas et al. (2020) in South Africa and Chola et al. (2023) in Zambia. The issue of ‘you are too young to seek contraceptives’ described in the South African study and confirmed in this study is concerning, given the provision for access to contraceptives for adolescents above the age of 12 years without parental consent (*Children’s Act of 2005*). This negative attitude from healthcare workers and community members could imply a need for awareness of adolescent girls’ right to access contraceptives. In addition, the negative attitudes could mean that there is a need for health workers to understand how these negative attitudes hinder adolescents’ use of contraceptives. Similarly, the negative perceptions of contraceptive use were attributed to the influence of parents whom the adolescents ‘feared’. This fear is explained by Participant 12 who said: ‘fear of parents finding out, that they might be intimate’. The study by Chola et al. (2023) in Zambia also draws similar conclusions that adolescents fear parents, thus discouraging contraceptive use.

### Limitations and recommendations

The study found that adolescent girls were influenced by male partners to use contraceptives as they did not want to have children. However, the study design did not include data collection from any men. This lack of inclusion of men in the study design limits the study findings, which could have been enriched by exploration of the influence of men from the experiences of men themselves. The study also found that adolescent girls had a positive perception of contraceptives because they wanted to prevent the financial burden on the families they came from. Findings from other studies highlight the fact that financial constraints affected access to contraceptives, which was a hinderance to contraceptive use. Given the contrast in findings, it is recommended that additional studies examine how financial constraints affect the use of contraceptives among adolescents. The issue of negative attitudes from healthcare workers and community members in a context where there are enabling policies promoting free contraceptive use without parental consent also needs urgent attention by policymakers to raise awareness of such policies and mitigate the negative effects of attitudes on contraceptive use.

## Conclusion

The study sought to describe the perceptions of adolescent girls regarding the use of contraceptives in Ekurhuleni. The study found that adolescent girls have positive and negative perceptions regarding the use of contraceptives. The findings from the study are important in addressing the low number of adolescent girls who use contraceptives leading to unintended pregnancies in Ekurhuleni. The study described the critical issue of negative attitudes of healthcare workers and community members, which have effects on the use of contraceptives and recommends the need to address such issues through programmes targeting adolescents. Moreover, a different insight on how financial constraints in families elicited positive perceptions of contraceptives is described, which could be explored further in other contexts to understand its effect on contraceptive use.
